# Modulation of Lipogenesis and Glucose Consumption in HepG2 Cells and C2C12 Myotubes by Sophoricoside

**DOI:** 10.3390/molecules181215624

**Published:** 2013-12-13

**Authors:** Chongming Wu, Hong Luan, Shuai Wang, Xue Zhang, Ran Wang, Lifeng Jin, Peng Guo, Xi Chen

**Affiliations:** 1 Pharmacology and Toxicology Research Center, Institute of Medicinal Plant Development, Chinese Academy of Medical Sciences and Peking Union Medical College, Beijing 100094, China; E-Mails: wucm1979@gmail.com (C.W.); xfxqe932077826@163.com (H.L.); zhuizhirun@163.com (S.W.); zxkuaile@163.com (X.Z.); 2 Zhengzhou Tobacco Research Institute of CNTC, Zhengzhou 450001, China; E-Mails: wangranljj2010@163.com (R.W.); jin_lf@126.com (L.J.)

**Keywords:** sophoricoside, glucosidase, glucose consumption, lipogenesis, lipolysis, AMPK

## Abstract

Sophoricoside, an isoflavone glycoside isolated from *Sophora japonica* (Leguminosae), has been widely reported as an immunomodulator. In this study, the effects of sophoricoside on lipogenesis and glucose consumption in HepG2 cells and C2C12 myotubes were investigated. Treatment with sophoricoside at concentrations of 1–10 μM inhibited lipid accumulation in HepG2 cells in a dose-dependent manner. At the same concentration range, no effect on cell viability was observed in the MTT assay. Inhibition of lipogenesis was associated with the downregulation of SREBP-1a, SREBP-1c, SREBP-2 and their downstream target genes (FAS, ACC, HMGR) as revealed by realtime quantitative PCR. The lipid-lowering effect was mediated via the phosphorylation of AMPK. Further investigation of the activities of this isoflavone showed that sophoricoside has the capability to increase glucose uptake by C2C12 myotubes. It also effectively inhibited the activities of α-glucosidase and α-amylase *in vitro* and remarkably lowered postprandial hyperglycaemia in starch-loaded C57BL6/J mice. These results suggest that sophoricoside is an effective regulator of lipogenesis and glucose consumption and may find utility in the treatment of obesity and type 2 diabetes.

## 1. Introduction

Type 2 diabetes (T2DM) is becoming a serious threat to human health in all parts of the World, placing an enormous burden on national healthcare systems, particularly in developing countries [1,2]. Modernized lifestyles featured by over-nutrition and less-exercise stimulate the increase in the prevalence of obesity and associated metabolic disorders including T2DM. In T2DM, insulin action is reduced, resulting in decreased insulin-stimulated glucose uptake. Many studies have shown that the over accumulation of lipids in liver is a key cause for the development of insulin resistance [3,4]. Decreasing lipid accumulation in liver through inhibition of lipogenesis or stimulation of lipolysis is therefore beneficial for the prevention and treatment of T2DM. Currently, studies on obesity and diabetes focus on discovering natural functional molecules that have the capability of suppressing the fat accumulation in liver tissues. For example, resveratrol [5], cordycepin [6], and chlorogenic acid [7] suppress lipogenesis and fat accumulation.

Sophoricoside is an isoflavone glycoside isolated from *Sophora japonica* (Leguminosae), a Traditional Chinese Medicine known to possess hemostatic properties, anticancer, anti-oxidation, anti-obesity and anti-hyperglycemic effects [8,9,10,11]. Previous investigations have demonstrated several biological effects of sophoricoside, such as estrogenic activity [12], anti-oxidation [13], anti-inflammation [14], stimulation of osteoblast proliferation [15], and immunomodulative activity [16]. Apart from these, there is no report about the lipid and glucose modulating activities of sophoricoside. In the present work, we investigated the effects of sophoricoside on lipid accumulation and glucose consumption in HepG2 cells and C2C12 myotubes, to find potential utility of sophoricoside in the prevention and treatment of obesity and type 2 diabetes.

## 2. Results and Discussion

### 2.1. Sophoricoside Inhibited Lipid Accumulation in HepG2 Cells

**Figure 1:**
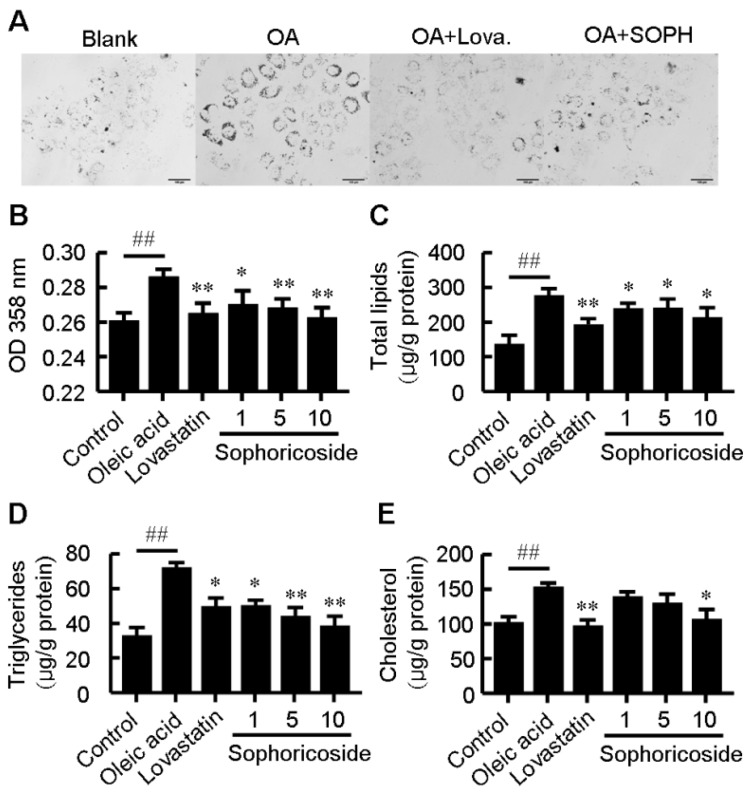
Effect of sophoricoside on lipid accumulation. HepG2 cells were treated with sophoricoside (in μM as indicated) or lovastatin (10 μM) in DMEM containing oleic acid (100 nM) or with serum-free DMEM alone (blank) for 24 h. (**A**) Typical pictures of Oil Red O staining. Bar = 100 μm; (**B**) The OD 358nm after Oil Red O staining; (**C**–**E**) Intracellular levels of total lipids (**C**) triglyceride; (**D**) and total cholesterol; and (**E**). Values represent mean ± SD. Results are representative of three different experiments with *n* = 3. ^##^ *p <* 0.01 *versus* blank group, * *p* < 0.05, ** *p* < 0.01 *versus* oleic acid group. OA: oleic acid; Lova: lovastatin; SOPH: sophoricoside.

**Figure 2:**
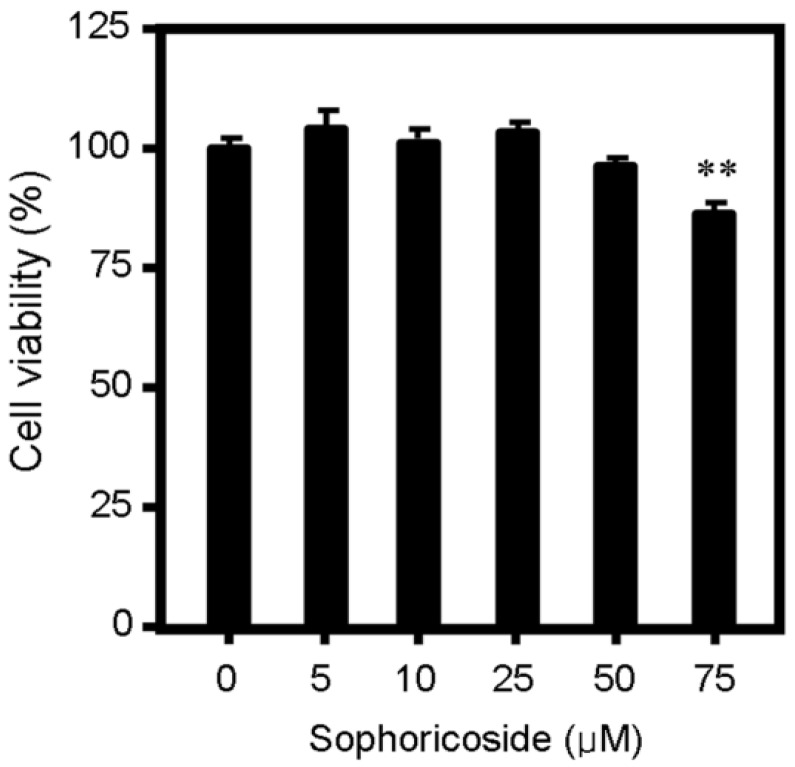
Effect of sophoricoside on cell viability as determined by an MTT assay. The inhibition on cell viability was expressed as a percentage of viable cells in experimental wells relative to control (0) wells. Values represent mean ± SD. Results are representative of three different experiments with *n* = 8. ** *p* < 0.01 *versus* control (0) group.

To evaluate the effect of sophoricoside on lipid metabolism, oleic acid (OA)-elicited neutral lipid accumulation in HepG2 cells was used and the intracellular lipid content was determined by Oil Red O staining and specific kits for cellular total lipids, total cholesterol and triglyceride. As shown in Figure 1, supplementation with OA significantly increased lipid accumulation in HepG2 cells. Treatment with sophoricoside decreased OA-elicited neutral lipid accumulation (Figure 1A,B) as well as intracellular contents of total lipids (Figure 1C), triglyceride (Figure 1D) and total cholesterol (Figure 1E) in a dose-dependent manner. This inhibitory effect on lipid metabolism was independent of the cytotoxic effect of sophoricoside on HepG2 cells, which was observed starting at a higher concentration (75 μM) in MTT assay (Figure 2).

### 2.2. Sophoricoside Decreased the Transcription of Lipogenesis-Related Transcription Factors and Their Target Genes

**Figure 3:**
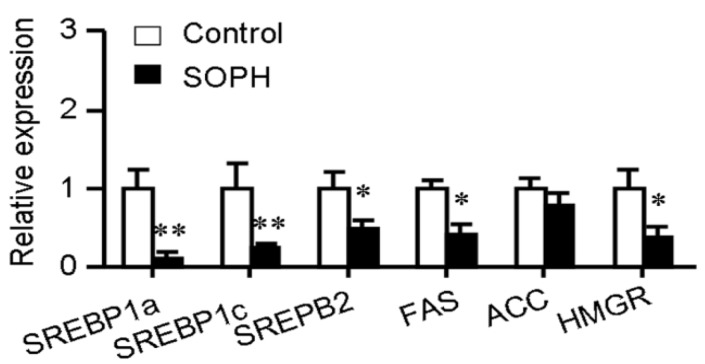
Effect of sophoricoside on SREBP-1a, SERBP-1c, SREBP-2, FAS, ACC and HMGR mRNA levels. Gene expression was quantified by realtime quantitative PCR analysis. The gene expression levels were normalized to β-actin mRNA levels. Values represent mean ± SD. Results are representative of 3 independent experiments with *n* = 3. * *p* < 0.05, ** *p* < 0.01 *versus* control group. SOPH: sophoricoside.

The synthetic processes of cholesterol and fatty acids are both controlled by a common family of transcription factors designated sterol regulatory element-binding protein (SREBPs) [17,18]. The mammalian genome encodes three SREBP isoforms named SREBP-1a, SREBP-1c and SREBP-2. SREBP-1a is a potent activator of all SREBP-responsive genes, including those that mediate the synthesis of cholesterol (3-hydroxy-3-methylglutaryl coenzyme A reductase (HMGR)), and fatty acids (fatty acid synthase (FAS) and acetyl-CoA carboxylase (ACC)) while the roles of SREBP-1c and SREBP-2 are more restricted. SREBP-1c preferentially enhances transcription of genes required for fatty acid synthesis while SREBP-2 preferentially activates cholesterol synthesis [17]. Quantitative realtime PCR showed that treatment with sophoricoside (10 μM) significantly decreased the expression of SREBP-1a, SREBP-1c and SREBP-2 transcription factors. The expression of FAS and HMGR were also reduced after sophoricoside treatment (Figure 3). These data suggested that sophoricoside suppresses both cholesterol and triglyceride synthesis.

### 2.3. Sophoricoside Increases the Activity of AMPK

AMPK is a key regulator of lipid metabolism, imposing profound influence on lipid oxidation, synthesis, and storage [19,20]. AMPK activation turns on ATP-generating mechanisms such as lipid oxidation while switches off energy-consuming processes like triglyceirde and protein synthesis [19,21]. The phosphorylation at threonine (Thr-172) on the alpha-subunit of AMPK has been deemed as an index of activation of this kinase which in turn promotes the phosphorylation and inhibition of ACC. Recent studies have demonstrated that AMPK can directly phosphorylate and inhibit SREBP activity to attenuate hepatic steatosis [22]. AMPK has now been proposed as a major therapeutic target for obesity and obesity-linked metabolic disorders including diabetes.

**Figure 4:**
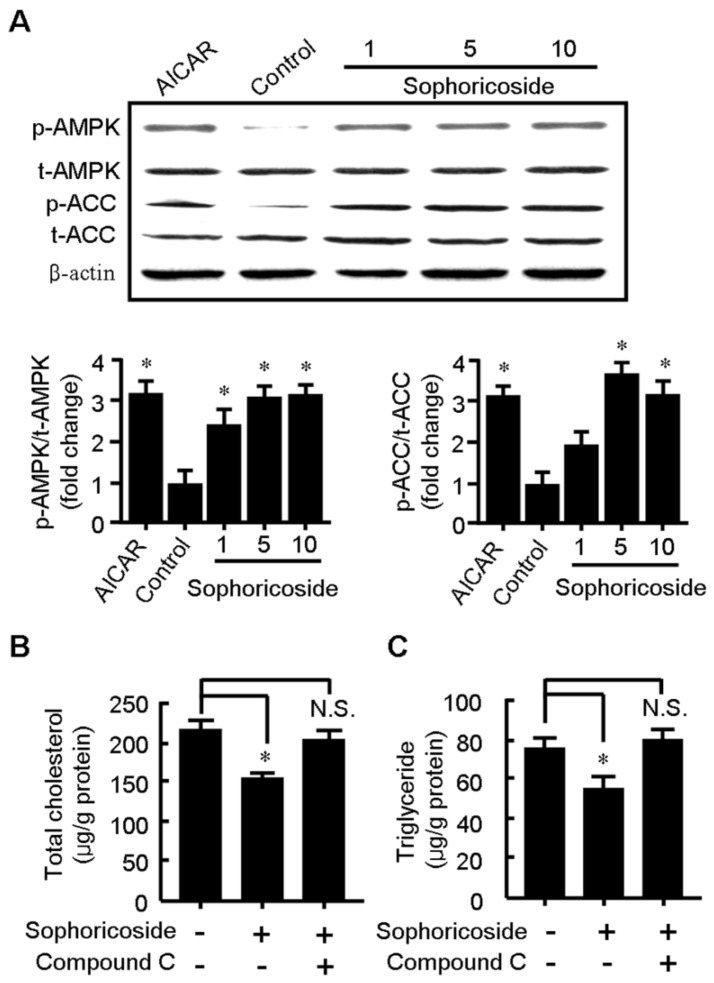
Sophoricoside increased the phosphorylation levels of AMPK and ACC (**A**) while AMPK inhibitor compound C substantially abolished the lowering effect of sophoricoside on the increased total cholesterol; (**B**) and triglyceride; and (**C**) elicited by oleic acid. The histograms represent the ratios of phospho-AMPK/total AMPK and phospho-ACC/total ACC, which represents the activation of AMPK. These values were obtained by quantification of the intensity of each band of the western blot. HepG2 cells were treated with either sophoricoside (indicated in 4A or 10 μM in 4B and 4C) or sophoricoside + compound C (40 μM) for 24 h. Values represent mean ± SD. Results are representative of three different experiments with *n* = 4. * *p* < 0.05 *vs.* control group or as indicated.

The protein levels of AMPK and its phosphorylated form (phospho-AMPK), which indicates the activation of AMPK, were evaluated by western blot. To confirm the activation of AMPK, the expression levels of ACC protein and its phosphorylated form (phospho-ACC) were simultaneously examined. As displayed in Figure 4A, the sophoricoside remarkably enhanced the levels of the activated form of AMPK (phospho-AMPK) and ACC (phosphor-ACC), which indicates the activation of AMPK by sophoricoside. Importantly, the lipid-lowering effect of sophoricoside was substantially abolished when cotreated with compound C, a well-known AMPK inhibitor (Figure 4B), suggesting that AMPK plays a key role in sophoricoside-mediated lipid-lowering effect.

### 2.4. Sophoricoside Stimulates Glucose Uptake by C2C12 Myotubes

**Figure 5:**
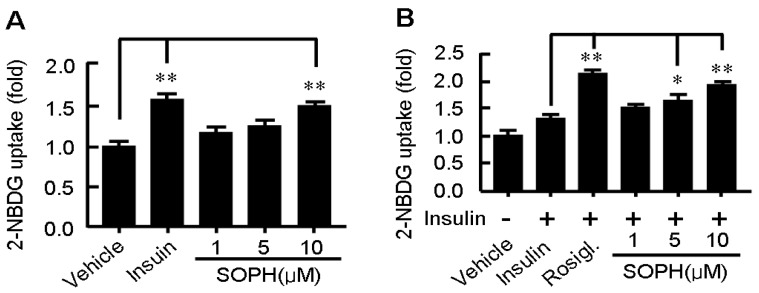
Effect of sophoricoside on glucose uptake by C2C12 myotubes in absence (**A**) or presence of insulin (100 nM); and (**B**) Differentiated C2C12 myotubes were incubated with the serum-free DMEM containing 2-NBDG (10 μM) and sophoricoside in the presence or absence of insulin for 12 h. Then cells were scraped out and the amount of 2-NBDG taken up by the cells was measured by determining the fluorescence intensities at Ex/Em = 475 nm/550 nm. Values represent mean ± SD. Results are representative of 3 different experiments with *n* = 3. * *p* < 0.05, ** *p* < 0.01. SOPH: sophoricoside.

We also found that sophoricoside activated glucose consumption in a dose-dependent manner in C2C12 myotubes. The effect of sophoricoside on glucose uptake was assessed by NBD-glucose (2-NBDG) uptake assay. Treatment with sophoricoside for 12 h increased 2-NBDG uptake by C2C12 myotubes in a dose-dependent manner (Figure 5A). The efficacy of sophoricoside (10 μM) was comparable to insulin (0.1 μM), suggesting a potent activity of sophoricoside in stimulating glucose uptake by myotubes. Additionally, sophoricoside also showed an obvious synergistic effect with insulin (Figure 5B). This interesting *in vitro* effect of sophoricoside needs to be followed by a detailed investigation to identify the factors directly responsible for the observations made here. Many studies have shown that activation of AMPK significantly increases glucose uptake in muscle cells. This glucose consumption-related kinase was activated by sophoricoside after 2 h of exposure. Irrespective of the activation state of AMPK, the kinase AKT and the translocation of GLUT1 and GLUT4 in the presence of sophoricoside should be studied more thoroughly.

### 2.5. Sophoricoside Inhibits the Activities of α-Glucosidase and α-Amylase *In Vitro* and *In Vivo*

**Figure 6:**
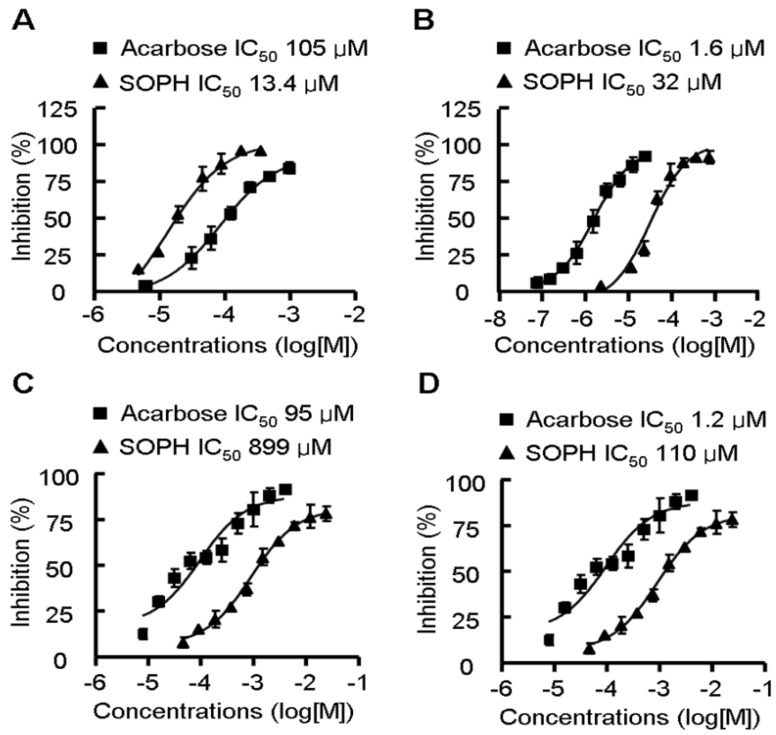
Inhibitory activity of sophoricoside (SOPH) on α-glucosidase from *S. cerevisiae* (**A**) *Rhizopus sp.*; (**B**) rat intestines; (**C**) on α-amylase from rat intestines; and (**D**) Inhibitory effect was determined using -nitrophenyl-α-glucopyranoside (PNPG) and starch as a substrate, respectively. Acarbose was used as positive control. Data were plotted and the IC_50_ values were calculated by GraphPad Prism 4 software [25]. Each value is expressed as mean ±S.D. of triplicate experiments. SOPH: sophoricoside.

**Figure 7:**
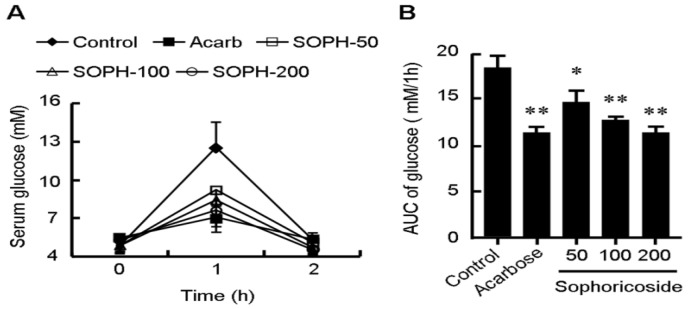
Blood glucose level. (**A**) area under curve (AUC); and (**B**) after dministration of sophoricoside (SOPH) in starch-loaded mice. Sophoricoside (50, 100 and 200 mg/kg), acarbose (100 mg/kg), and control (distilled water) were co-administered orally with starch (4 g/kg). Each value is expressed as mean ±S.D. of 12 mice. * *p* < 0.05, ** *p* < 0.01 *vs.* control. SOPH: sophoricoside.

Management of the postprandial blood glucose is an important method to deal with diabetes and now attracting more and more attentions. Amylase and glucosidase are two key saccharide hydrolysing enzymes responsible for increase of the postprandial blood glucose [23,24]. We further investigated the inhibitive effect of sophoricoside on the activity of α-glucosidase and α-amylase *in vitro* and *in vivo*. As shown in Figure 6, sophoricoside inhibited the activity of α-glucosidase and α-amylase in a dose-dependent manner. The IC50 values of sophoricoside on α-glucosidase from *S. cerevisiae*, Rhizopus sp. and rat intestines were 13.4 μM, 32 μM and 899 μM, respectively. The IC50 value of sophoricoside on α-amylase from rat intestines was 110 μM. The *in vivo* effect of sophoricoside on postprandial glycaemia was evaluated in starch-loaded C57BL6/J mice (Figure 7). Oral administration of a single dose of sophoricoside significantly decreased the elevated blood glucose level at 1 h (by 26.49%, 32.67% and 39.34% for 50, 100 and 200 mg/kg, respectively). The efficacy of 200 mg/kg of sophoricoside was comparable to that of 100 mg/kg of acarbose (39.34% *vs.* 43.52%). These results suggest that sophoricoside is an effective inhibitor of α-glucosidase and α-amylase *in vitro* and *in vivo*.

## 3. Experimental

### 3.1. Materials

HepG2 and C2C12 cells, which originated from the American Type Culture Collection (ATCC) (Manassas, VA, USA), were obtained from the Peking Union Medical College (Beijing, China). Sophoricoside, with a purity of 98% as determined by HPLC, was purchased from Forever-Biotech Co. Ltd (Shanghai, China). A voucher specimen has been deposited in Institute of Medicinal Plant Development, Chinese Academy of Medical Sciences & Peking Union Medical College (NO. SOPH20120706). Dulbecco’s modified Eagle’s medium (DMEM), 3,5-dinitrosalicylic acid, 4-nitrophenyl-α-D-glucopyranoside (PNPG), 2-deoxy-2-[(7-nitro-2,1,3-benzoxadiazol-4-yl)amino]-D-glucose (2-NBDG), 3-(4,5-dimethylthiazol-2-yl)-2,5-diphenyltetrazolium bromide (MTT), α-glucosidase from Saccharomyces cerevisiae, rat intestinal acetone powder were procured from Sigma-Aldrich, Inc. (St Louis, MO, USA). α-Glucosidase from Rhizopus sp. was sourced from Gold Wheat Biotech, Inc. (Shanghai, China). Antibodies for total AMP-activated protein kinase α (AMPKα), phosphospecific-AMPKα(Thr172), total acetyl-CoA carboxylase (ACC), phosphospecific-ACC(Ser 79) and total β-actin antibodies were obtained from Cell Signaling Technology (Beverly, MA, USA).

### 3.2. Cell Culture

C2C12 myoblasts were mantained in DMEM supplemented with 10% FBS at 37 °C and 5% CO_2_. To induce differentiation, media was replaced with DMEM containing 2% horse serum when the cells reached confluence. The C2C12 cells were kept in this differentiating medium for 5 days, which allowed cells to be fully differentiated and used in the following experiments. HepG2 cells were cultured in DMEM medium containing 10% fetal bovine serum (FBS) at 37 oC and 5% CO2. Before treatment, cells were kept in serum-free DMEM for 12 h then incubated with the indicated concentration of sophoricoside or with lovastatin (10 μM) in DMEM containing oleic acid (100 nM) for 24 h. The blank group was incubated with serum-free DMEM alone. Oil red O staining was performed as previous reported [26] and the intracellular contents of total lipid, total cholesterol and triglyceride were determined by kits according to manufacturer’s instructions.

### 3.3. MTT Assay

HepG2 cells were cultured in a 24-well plate. After reaching confluence, the cells were incubated for 48 h in the presence of sophoricoside. Subsequently, the culture medium was removed and replaced by 500 μL of fresh culture medium containing 10% sterile filtered MTT. After 3 h, the insoluble formazan crystals were dissolved in 500 μL/well isopropanol and absorbance was measured at 570 nm, using the 630 nm reading as a reference. The inhibition of growth due to sophoricoside was expressed as a percentage of viable cells in experimental wells relative to control wells.

### 3.4. Western Blot

HepG2 cells were lysed in lysis buffer containing 10% glycerol, 1% Triton X-100, 135 mM NaCl, 20 mM Tris (pH 8.0), 2.7 mM KCl, 1 mM MgCl2, and protease and phosphatase inhibitors (0.5 mM PMSF, 2 μM pepstatin, and 2 μM okadaic acid). Aliquots of samples were subjected to SDS-PAGE followed by transfer to polyvinylidene difluoride (PVDF) membranes (Amersham Pharmacia, Uppsala, Sweden). Immunoblotting was performed using respective antibodies (1:1000). Following incubation with horseradish peroxidase-conjugated secondary antibody (Sigma-Aldrich, Shanghai, China), proteins were detected with ECL plus kits (Amersham, Piscataway, NJ, USA).

### 3.5. Quantitative Real-Time PCR

The mRNA levels of lipid metabolism-related genes were determined by real-time quantitative PCR. Total RNA extraction, cDNA synthesis and quantitative PCR assays were performed as described previously [27]. Samples were cycled 40 times using a Fast ABI-7500 Sequence Detector (Applied Biosystems, Foster City, USA). ABI-7500 cycle conditions were as follows: 5 min at 95 °C followed by 40 cycles of 15 s at 95 °C, 30 s at 60 °C and 30 s at 72 °C. Cycle threshold (CT) was calculated under default settings for real-time sequence detection software (Applied Biosystems). At least three independent biological replicates were performed to check the reproducibility of the data. The gene-specific primers used for quantitative PCR are listed in Table 1.

**Table 1:** Primers used in realtime quantitative PCR analysis.

Name	Forward (5'-3')	Reverse(5'-3')
SERBP-1a	tgctgaccgacatcgaagac	ccagcatagggtgggtcaa
SREBP-1c	ccatggatgcactttcgaa	ccagcatagggtgggtcaa
SREBP-2	ctgcaacaacagacggtaatga	ccattggccgtttgtgtcag
FAS	CGGTACGCGACGGCTGCCTG	GCTGCTCCACGAACTCAAACACCG
ACC	TGATGTCAATCTCCCCGCAGC	TTGCTTCTTCTCTGTTTTCTCCCC
HMGR	ggacccctttgcttagatgaaa	ccaccaagacctattgctctg
β-actin	CCTGGCACCCAGCACAAT	GCCGATCCACACACGGAGTACT

### 3.6. Glucose Uptake Assay

Glucose uptake assay was performed as previously reported [28]. Briefly, differentiated C2C12 myotubes were incubated with the serum-free DMEM containing the fluorescent glucose analog 2-NBDG (10 μM) and sophoricoside (1, 5 or 10 μM) in the presence or absence of insulin (100 nM). After incubation for 12 h, medium was removed and cells were washed with phosphate-buffered saline (PBS) twice. Cells were scraped out in 1 mL of PBS and transferred into 5 mL polystyrene round-bottom tubes (BD Falcon) and kept at 4 °C. The amount of 2-NBDG taken up by the cells was measured by determining the fluorescence intensities at Ex/Em = 475 nm/550 nm using a Tecan Infinite M1000Pro Microplate Reader (TECAN Group Ltd, Shanghai, China).

### 3.7. α-Glucosidase and α-Amylase Inhibition Assay

Inhibition of α-glucosidase and α-amylase activities was performed according to the chromogenic method described previously [29,30], using PNPG and starch as substrate, respectively. Acarbose was used as positive control. The inhibitory activity was expressed as percentage inhibition, equation (1). The IC_50_ values were calculated by GraphPad Prism 4 software [25].

Inhibition (%) = [(Abs_Control_ − Abs_Samples_)/Abs_Control_] × 100 (1)

### 3.8. Animal Experiment

Male C57BL6/J mice (20–22 g each) were obtained from the Institute of Laboratory Animal Science, Chinese Academy of Medical Sciences, Beijing, China. The study was carried out according to the “Principles of Laboratory Animal Care” [World Health Organization (WHO) Chronicle, 1985] and approved by the Animal Ethics Committee of Peking Union Medical College. A standard pellet diet and water were given *ad libitum*. Animals were maintained under a constant 12 h light and dark cycle and an environmental temperature of 21–23 °C.

Animals were randomly divided into five groups with twelve mice in each group. Prior to the experiment, animals were fasted for eight hours then treated with corresponding agents orally. After another 15 min, each mouse was given 4 g/kg of starch (oral gavage). Blood glucose levels at 0, 1 and 2 h after starch administration were determined by a blood glucose meter (ACCU-CHEK Active, Roche, city, country). The control group received distilled water only, whereas acarbose group received acarbose (100 mg/kg, oral gavage). SOPH groups were treated with sophoricoside (50, 100 and 200 mg/kg respectively, *p.o.*).

### 3.9. Statistics Analysis

The results were expressed as mean ± SD. A one-way analysis of variance (ANOVA) was done using the SPSS 13.0 software [31]. Significance was accepted at *p* < 0.05.

## 4. Conclusions

This work demonstrated that sophoricoside isolated from *Sophora japonica* (Leguminosae) is able to inhibit lipid accumulation in HepG2 cells and stimulate glucose consumption in C2C12 myotubes. Decreasing transcription of key lipogenic genes and activating AMPK may involved in the regulating effect of sophoricoside on lipid and glucose metabolism. These results suggest that sophoricoside is an effective regulator of lipogenesis and glucose consumption and may find utility in the treatment of obesity and type 2 diabetes.
